# Patient satisfaction with cataract surgery

**DOI:** 10.1186/1755-7682-1-22

**Published:** 2008-10-25

**Authors:** Ehab I Wasfi, P Pai, Alaa A Abd-Elsayed

**Affiliations:** 1 Eye Department, Assiut University Hospital, Assiut, Egypt; 2 Eye Department, Barrow General Hospital, Barrow in Furness, UK; 3 Department of Public Health and Community Medicine, Faculty of Medicine, Assiut University, Egypt

## Abstract

**Introduction:**

Measuring the patient satisfaction is a very important issue that will help very much in improving the service provided to patients and improve the level of satisfaction.

**Aim:**

To evaluate patient satisfaction with the cataract surgery service and identify any areas for improvement, determination of patient satisfaction with referral, out-patient consultation, pre-assessment clinic, surgery and post-operative care, also to report patients' comments relating to improvement in service provision.

**Methodology:**

A retrospective study was undertaken for 150 patients underwent cataract surgery at Barrow General Hospital, UK, the survey sample was by postal questionnaires. We collected our data from the theatre lists for a period of 4 month.

**Results:**

This study included 150 patients; the response rate was (72%) 108 patients, Most patients were referred from their general practitioner 86.1%, 93 (86.1%) patients were happy with the time interval from seeing their GP to eye clinic. In the eye out patient department many factors significantly affected the level of patient satisfaction, in general the more information provided for the patient the more the satisfaction.

**Conclusion:**

Patient satisfaction is on important health outcome old understanding both the domains of satisfaction as well as their relative importance to patients is necessary to improve the overall quality of patient care. Meeting the doctor, presenting all relevant information and giving printed information are very important factors in improving the patient's satisfaction with cataract surgery.

## Introduction

Patient satisfaction is a very subjective concept which is difficult to measure. Surveying the experiences and views of patients can provide useful information. Achievement of service provision is a good indicator of patient satisfaction.

## Methodology

A retrospective study was undertaken for 150 patients underwent cataract surgery at Barrow General Hospital, UK, the survey sample was by postal questionnaires. We collected our data from the theatre lists for a period of 4 month.

The response rate was 72%. Patients were surveyed as to the route of their referral, their satisfaction of the time interval before seeing the ophthalmologist, with the eye out patient clinic service including any information handout given, the explanation before doing the preoperative assessment and the cataract operation. Also patients were asked to rate their views regarding the waiting list time before the surgery and if there were any cancellation or deferral of the operation.

We evaluated also the anesthetic service given to all patients, and the care given to the patient during and after the surgery. The surgeries were performed by 3 consultants and 3 junior staff. Phacoemulsification technique was used in 98% of the cataract extractions.

Each patient completed an extensive self administered subjective questionnaire which was sent to them with a return envelops. Medline literature search was performed. The Statistical Package for the Social Sciences, version 13 (SPSS Inc, Chicago, IL, USA) was used for statistical analysis.

## Results

This study included 150 patients; the response rate was (72%) 108 patients.

There was no response from 42 patients either because they are physically or intellectually disabled. 39.8% were male and 35% were female (Table 1), the majority of the patients were between 65–84 years (75%) (Table 1). Most patients were referred from their general practitioner 86.1% (Table 1).

**Table 1:** Patient satisfaction survey-cataract surgery

**Question**	**Number**	**%**
**Consultant:**		
Pink	37	34.3
Yellow	36	33.3
Blue	35	32.4
**Age groups:**		
<45	1	0.9
45-	2	1.9
55-	9	8.3
65-	37	34.3
75-	44	40.7
≥85	15	13.9
**Gender:**		
Male	43	39.8
Female	38	35.2
Not stated	27	25
**GP referral to clinic**	93	86.1
**Hospital referral**	2	1.8
**Self presentation**	1	0.9
**Eye Clinic attendee**	7	6.5
**Saw specialist privately**	5	4.7

Most patients (86.1%) were happy with the time interval from seeing their GP to eye clinic (Table 1). About 67.6% of patients were seen within 6 month of their General Practitioner appointment. In the day of their eye clinic appointment 43 (39.8%) of them were seen within 30 minutes of their clinic appointment.

**Figure 1:**
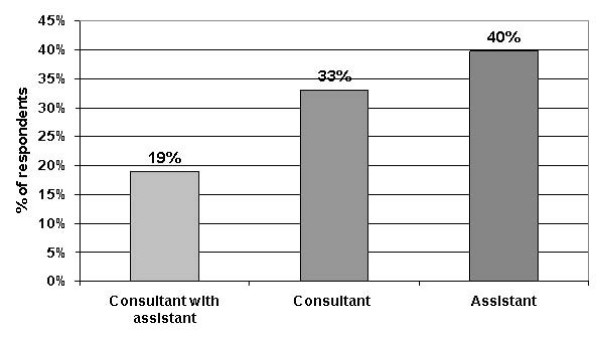
Out-patient consultation.

In the eye out patient department 36 (33%) patients were seen by a consultant while 43 (39.8%) patient were seen by his assistant (Figure 1). In the eye out patient department many factors significantly affected the level of patient satisfaction, in general the more information provided for the patient the more the satisfaction, shown in Table 2.

**Table 2:** Visit to the eye clinic

**Question**	**No. (%)**	**No. of satisfied patients (%)**	***P *value**
Doctor introduced him/herself	86 (79.6%)	77(71.3%)	0.03*
Doctor made patient reasonably relaxed	100(92.6%)	89(82.4%)	0.01**
Patient felt doctor gave clear explanation	91(84.3%)	81(75%)	0.002***
Patient understood everything	99(91.7%)	95(88%)	0.000***
Patient invited to ask questions	76(70.4%)	75(69.4%)	0.02*
Patient given enough time to discuss everything	93(86.1%)	91(84.3%)	0.004**
Patient understood what would happen next	93(86.1%)	89(82.4%)	0.000***
Doctor discussed with patients:			
Benefits of surgery	81(75.0%)	79(73.1%)	0.04*
Possible risks of surgery	54 (50.0%)	60(55.6%)	0.02*
Alternatives	14(13.0%)	11(10.2%)	0.007**
Anesthesia	76(70.4%)	56(51.8%)	0.09
How patient would feel immediately post-surgery	65(60.2%)	63(58.3%)	0.01*
Length of hospital stay	82(75.9%)	59(54.6%)	0.11
How patient would feel long-term post-surgery	71 (65.7%)	55(50.9%)	0.03*
Patient felt doctor had provided all necessary information	92(85.2%)	88(81.5%)	0.004**
Doctor gave printed information	85(78.7%)	96(88.9%)	0.001**

* P < 0.05; ** p < 0.01; *** p < 0.001

In the pre assessment clinic the rates were as in Table 3. 92 (85.2%) patients had less than 6 month from eye clinic appointment to the day of surgery.

**Table 3:** Pre-assessment clinic

**Question**	**No. (%)**
Attended clinic	94(87%)
Received booklet	96(88.9%)
Patient read booklet	99(91.7%)
Understood booklet	94(87%)
Patient had no worries after reading booklet	84(77.8%)
Explanation re: tests	82(75.9%)
Understood procedures during admission	85(87%)

Majority of patients (94.4%) were admitted and operated upon on the planned date, operation re-scheduled within 1 month for 4 patients and cancelled for 2 patients. Local anesthetic was given in 97 (95.3%) patient; the anesthetists introduced him or her self and explained clearly the procedure in all patients (100%). The nurse held patient hands in 81 (82.6%) patient which reassured 69 (85.2%) patients of them.

In the eye ward following surgery whether it is a day case or inpatient the patients rated the care and the services offered from the ward staff as in (Table 4).

**Table 4:** Ward after operation

**Question**	**No. (%)**
Patient rated care as "very good" or "good"	98(90.7%)
Day case	95(88%)
Length of hospital stay was "right for me"	99(91.7%)
Patient given ample notice re: discharge	103(95.3%)
Given information re: self-care post-discharge	105(97.2%)
Information covered all/most things	102(94.4%)
Staff assessed ability to manage at home	81(75%)
Given contact details	101(93.5%)
Did not require pain relief once home	85(78.7%)

**Figure 2:**
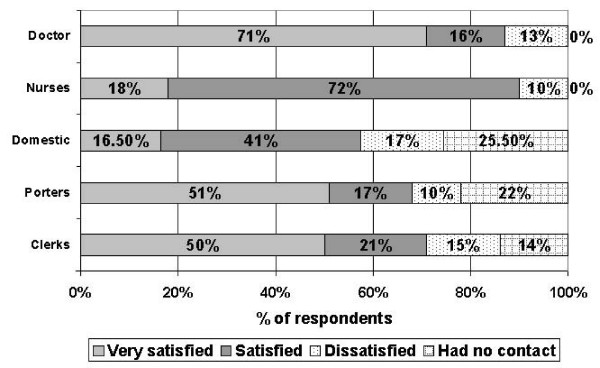
Patient satisfaction with staff.

Then patients felt that improvement to cataract services could be made in following areas as in (Table 5). Patient satisfaction with staff was as in (Figure 2).

**Table 5:** Patients felt improvement could be made in the following areas

**Question**	**No. (%)**
The first clinic appointment	10(9.3%)
Pre-assessment clinic	9(8.3%)
Admission to Eye Ward	4(3.7%)
In theatre prior to operation	3(2.8%)
Operation	2(1.9%)
Discharge arrangements	2(1.9%)
Information to take home on discharge	5(4.6%)
Follow-up arrangements	8(7.5%)

We had 24 positive comments from the patients, while the negative comments were 19. The positive comments were praising the service and thanks to the friendly sympathetic and the professional staff from nurses, porters, clerks and doctors. Patients commented how much they felt relaxed and at ease this encouraged them to think about having the other eye done.

The negative comments stressed on the waiting time in the out patient clinic which lasted up to 2 hours in some patients, and I think for US to meet the NHS target we should encourage doctors to see the patients and patient information regarding the delay, other negative comments included lack of information about the post operative time needed for the patient to be able to read. Another patient was not happy to come 8.00 am in the 1^st ^post-operative day. One particular patient said that he was not happy when the doctor told him about the complications which may occur during or after surgery and he commented that a "frightening tactics" used by the hospital to decrease the number of patients going for surgery.

## Discussion

Patient satisfaction in cataract surgery is mediated by the difference in expectation and actual performance.

This paper is based on 108 patients who completed and sent the post operative questionnaire, the response rate was 72% while Conner-Spady et al. found 79% response rate [1].

There were no significant differences in age, sex, length of time waited between responders who did and did not complete the questionnaire. Cataract operation was rescheduled for 4 patients and cancelled for 2 patients.

Most of our patients (85%) had less than 6 month in the waiting list for their cataract surgery. About 89% of them felt satisfied with this interval while Conner-Spady et al. [1] reported that satisfied patients waited on average of 3–4 months compared with approximately 7 months for unsatisfied patients, while other studies [2,3] have shown that cataract patients are generally accepting of wait times of 3 months or less.

A Swedish study reported that approximately 90% of cataract patients chose to wait longer rather than go to another hospital with a shorter waiting time [4]. Naumann et al. found that notifying patients of their expected waiting time is important and increase their perception of fairness and satisfaction [5].

In our study 40% of respondents waited less than 30 minutes in the out patient clinic (The Guide to NHS target is 100%).

We found in this study that the patient satisfaction significantly increases with meeting the doctor and knowing more information about the surgery, same was found by Elder and Suter [6] who found that it is important to meet the surgeon, know the advantages and disadvantages of possible treatments, the common risks and complications, the operative technique, and discussion of the rare risks of the operation. Comprehensive preoperative information causes little or no increase in overall patient anxiety [7-11].

Poor patient recall of verbal preoperative information is well documented [12,8,9,13] and most respondents wanted written preoperative information. A standard written information sheet may also be the best medium in which to mention rare complications, leaving time for the surgeon to verbally discuss the particular risks and postoperative expectations pertaining to that particular patient. In our study the patients' satisfaction was significantly increased by giving them printed information.

The overall patient satisfaction in our study from being listed for surgery to discharge was high > 85%, while Chet and McCluskey [14] who compared public and private patients priorities and satisfaction found that 90% of private patients were satisfied with the information they received regarding surgery, while in public sector 45% of patients wonted more information.

## Conclusion

Patient satisfaction is on important health outcome old understanding both the domains of satisfaction as well as their relative importance to patients is necessary to improve the overall quality of patient care. Meeting the doctor, presenting all relevant information and giving printed information are very important factors in improving the patient's satisfaction with cataract surgery.

## Recommendations

• Implementation of system to convey reason for and degree of clinic delays.

• Reconsider the timing of first post-operative visit, as patient comments indicate that it can be very difficult to attend in the early morning.

• Providing all the relevant information for the patient prior to surgery.

• Ensure all patients receive written information at clinic visit, to minimize patient anxiety.

• Contact scheme for patient information/support.

## Competing interests

The authors declare that they have no competing interests.

## Authors' contributions

EW participated in the clinical work and the manuscript writing; PP participated in the clinical work and data collection and AAA-E participated in writing the final manuscript, data collection, data management and gave important clinical suggestions for patient care and management. All authors read and approved the final manuscript.
